# Factors contributing to the reduction in childhood stunting in Bangladesh: a pooled data analysis from the Bangladesh demographic and health surveys of 2004 and 2017–18

**DOI:** 10.1186/s12889-021-12178-6

**Published:** 2021-11-16

**Authors:** Pradeep Kumar, Rashmi Rashmi, T. Muhammad, Shobhit Srivastava

**Affiliations:** International Institute for Population Sciences, Mumbai, Maharashtra 400088 India

**Keywords:** Stunting, Decadal change, Under-5-y children, Bangladesh

## Abstract

**Background:**

Over the last two decades, Bangladesh has made progress in reducing the percentage of stunted children under age 5 years from 51% in 2004 to 31% in 2017. Such reduction has created a source for new research to understand its contributing factors. The present study aims to identify such crucial factors which contributed in reducing the percentage of under-five stunting status of children from 2004 to 2017–18.

**Methods:**

The study used data from the Bangladesh Demographic and Health Surveys (BDHS), conducted in 2004 and in 2017–18, focused on children under-5-years of age (U5). The sample sizes were *n* = 6375 children included in the 2004 survey and *n* = 8312 children included in the 2017–18 survey. Descriptive analysis and bivariate analysis were conducted for a general characterization of the samples. Logistic regression was used to find out the significant factors contributing to the prevalence of stunting among U5 children. Furthermore, the Fairlie decomposition technique was used to identify the crucial factors that contributed to the reduction of stunting.

**Results:**

The prevalence of stunting among U5 children has declined significantly, from 49.8 to 30.7% between the two survey periods (2004 and 2017–18). Estimates of decomposition analysis show that overall, the selected variables explained 50.6% of the decrease in the prevalence of stunting. Mother’s characteristics such as age at first birth, education level, working status and BMI (body mass index) status were the primary contributors of this change. Father’s characteristics, such as education explained 9% of this change.

**Conclusion:**

The results of the study highlight the importance of increasing maternal education and reducing inter-household wealth inequality to improve nutritional status of U5 children. In order to achieve further reduction in stunting, among U5 children in Bangladesh, this paper calls for policymakers to develop effective programs to improve maternal education, raise parental awareness of parents regarding children’s height and weight, and aim to significantly reduce inter-household inequalities.

## Background

Over the last two decades, different policies and programs have worked together to uplift the success of child survival across the globe [1–4]. Despite these improvements, deprivation from adequate nutrition along with recurrent illness among children has paved the way for a critical health issue [5–7]. Stunting, defined by the WHO [8] as “impaired growth and development that children experiences from poor nutrition, repeated infection and inadequate psychosocial simulation” is one of the grratest child health challenges which leaves a life-long mark on the individual [6, 7].

Globally, approximately 149 million children experience stunting before their fifth birthday [9]. The Global Overview Child Malnutrition Regional Trends Report of UNICEF shows that in Asia stunting still aflicts more than half of the Under-5 (U5) children (55%. More specifically, the situation of children in South Asian countries is alarming. Bangladesh is one such country which has experienced, throughout the decades, many ups and downs in the prevalence of stunted children. Being in a frequent natural disaster-prone area, the possibility of infectious diseases and inadequate nutrition are higher among the population [10, 11]. During the 1974 famine, the country experienced the highest (71%) Under-5 rates of stunting [12]. This has decreased to 31% in 2017 [13]. The country had made remarkable progress in reducing the prevalence of stunting, but it is still far from the 15% threshold marked by World Health Organization for an emergency situation in stunting for any country [14].

Extant research had repeatdly pointed out the prevalence of stunting in U5 children and its associated risk factors in Bangladesh [12, 15–20]. Studies have revealed that the effect of childhood stunting is long lasting and, in most cases, irreversible. Stunting is associated with lower mental capacity, slower cognitive development, and low school performance that will impact adult productivity. It is also related with an increase in mortality rates and an overall deteriotaon of the children’s health status [6, 21–25]. And these challenges can further topple the growth and development of a family, society and country as a whole.

Stunting develops at very early age due to unhealthy environments experienced by both mother and child [25, 26]. Previous literature in Bangladesh has linked the risk of under-five stunting with socio-demographic, healthcare services and nutrition-related factors [15, 19, 27, 28]. Although focusing on nutrition and recurrent diseases among children have helped in improving their health, there is the necessity to focus on the first and foremost proximal setting of a child: the family. A community-level cross-sectional study also revealed that over 85% of main caregivers in Bangladesh households are mothers who play an influential role in arranging and providing food for their children [29]. A study, using multilevel approach, has clearly shown that the risk of stunting was higher among children whose parents have the lower educational level and poorest wealth index, if the age of mother at the birth of the first child was below 20 years and if child’s age range was 36–47 months [16]. Furthermore, in Bangladesh research has highligthed the importance of improved water source and sanitation facility in a household towards child health [27, 30]. Moreover, even if the public health services are advanced, household wealth inequalityis strongly associated with childhood growth faltering [28]. Household wealth inequality measures the unequal economic distribution across the households which affects distinctly the food availability, access to health services, morbidity and mortality among the various sections of society. It was worth noting that children from the poorest household were thrice more likely to experience adverse stunting than those who reside in the wealthiest households. A cross country study has shown that, between 1997 and 2007, Bangladesh has recorded a fairly rapid progress against malnutrition among the low-and-middle-income countries [31] and has sparked interest about which contributing determinants of this reduction are.

Therefore, the main goal of this study is to identify the crucial factors that contributed to the reduction of stunting among U5 children in Bangladesh, between 2004 and 2017–18. The rationale for this analysis is threefold: i) Over the years, nutritional commitments at global platform have helped the countries across the world either to achieve their desired goal or to move one step forward without achieving fully. Such success or failure of programs help to modify the strategies and policies of the government. A country like Bangladesh which has observed many ups and downs in under-5 stunting status, there a study which can provide a clear picture of potential drivers is the need-of-the-hour. Although the past evidence shows the determinants of under-5 stunting status of Bangladeshi children, there is need for recent evidence which this study brings out.; ii), ample evidence shows the association of lower stunting status of children with the higher education and wealth status of family [32]. Studies have also pointed towards the key role of sanitation and demographic factors as the drivers of change. However, minimal evidence shows the role of the mother’s health on child stunting status, which have been explored in the present study; iii), with the limitations in nutrition-related information for under-five children in Bangladesh, this study pronounced to consider family (i.e. mother and father) and community related characteristics along with the household socio-economic and demographic factors. Thus, using two rounds of Bangladesh Demographic and Health Survey and Fairlie decomposition technique [33], this study explores the contributing factors of reduction in under-five stunting status in Bangladesh.

## Methods

### Data

This study utilized data from Bangladesh Demographic and Health Survey (BDHS), a nationally-representative survey conducted by the National Institute for Population Research and Training (NIPORT) of the Ministry of Health and Family Welfare [34, 35]. BDHS collected data on the nutritional status of children by measuring the height and weight of all children aged between 0 and 59 months in the selected households. The present study used two rounds of BDHS in 2004 and 2017–18 .

The sample for BDHS-2004 covered the entire population residing in private dwelling units in the country [35]. Administratively, Bangladesh is divided into six divisions. In turn, each division is divided into *zilas,* and each zila into *upazilas.* Each urban area in the *upazila* is divided into wards, and into *mahallas* within the ward; each rural area in the *upazila* is divided into *union parishads* (UP) and into *mouzas* within the Ups [35]. The urban areas were stratified into three groups, i) Standard metropolitan areas, ii) Municipality areas, and iii) Other urban areas. These divisions allow the country, as a whole, to be easily separated into rural and urban areas. For the 2001 census, subdivisions called enumeration areas (EAs) were created based on a convenient number of dwelling units. Because sketch maps of EAs were accessible, EAs were considered suitable to use as primary sampling units (PSUs) for the 2004 BDHS [35]. In each division, the list of EAs constituted the sample frame for the 2004 BDHS survey. The BDHS-2004 sample is a stratified, a multistage cluster sample consisting of 361 The primary sampling units (PSUs), were 122 in the urban areas and 239 in the rural areas [35]. After the target sample was allocated to each group area, according to urban and rural status, the number of PSUs was calculated in terms of an average of 28 completed interviews of eligible women per PSU (or an average of 30 selected households per PSU) [35].

The BDGS-2017-18 used a sampling frame from the list of enumeration areas (EAs) of the 2011 Population and Housing Census of the People’s Republic of Bangladesh, provided by the Bangladesh Bureau of Statistics (BBS). The primary sampling unit (PSU) of the survey is an EA with an average of about 120 households. The survey is based on a two-stage stratified sample of households. In the first stage, 675 EAs (250 in urban areas and 425 in rural areas) were selected with probability proportional to EA size. In the second stage of sampling, a systematic sample of an average of 30 households per EA was selected to provide statistically reliable estimates of key demographic and health variables for the country as a whole, for urban and rural areas separately [34]. All methods were performed in accordance with the relevant guidelines and regulations.

The effective sample size for the analysis was 6375 and 8312 U5 children in Bangladesh for the years 2004 and 2017–18, respectively. Only children with data for height and weight were included in the analysis.

### Variable description

#### Outcome variable

The outcome variable for the present study is stunting among U5 children in Bangladesh. Stunting was defined as height for age < − 2 standard deviation (SD) from the median of the WHO growth standards [36].

#### Explanatory variable

The explanatory variables include different individual, household, socio-economic and demographic factors which have been shown to influence stunting status of U5 children [15–17, 19]. The variables were divided into four sections, for analysis purposes: i) mother characteristics, ii) father characteristics, ii) child characteristics and iv) household characteristics.

#### Mother’s characteristics

Age at first birth was recoded as 0 = less than 18 years and 1 = 18 years or more, educational status was recoded as 0 = not educated and 1 = educated (completed primary education and above), working status was recoded as 0 = not working and 1 = working and low body mass index (BMI) was recoded as 0 = no (≥18.5) and 1 = yes (< 18.5).

#### Father’s characteristics

Educational status was recoded as 0 = not educated and 1 = educated (primary completed and above), working status was recoded as 0 = not working and 1 = working.

#### Child’s characteristics

Child’s age (in months) was recoded as 1 = 0–23, 2 = 23–47 and 3 = 48–59, sex was recoded as 1 = male and 2 = female, birth order was recoded as 1 = one, 2 = two, 3 = three and 4 = four and above.

#### Household characteristics

Protected water source [36] was recoded as 0 = no (Unprotected dug well, unprotected spring, cart with small tank/drum, tanker truck, and surface water (river, dam, lake, pond, stream, canal, irrigation channels), bottled water) and 1 = yes (Public taps or standpipes, tube wells or boreholes, protected dug wells, protected springs and rainwater collection, Piped household water connection located inside the user’s dwelling, plot or yard), improved toilet source [36] was recoded as 0 = no (flush or pour-flush to elsewhere, pit latrine without slab or open pit, bucket, hanging toilet or hanging latrine and no facilities or bush or field (open defecation) and 1 = yes (flush or pour-flush to piped sewer system, septic tank or pit latrine, ventilated improved pit latrine, pit latrine with slab and composting toilet), wealth index was recoded as 1 = poorest, 2 = poorer, 3 = middle, 4 = richer and 5 = richest. The variable of wealth status was created using the information given in the survey. Households were given scores based on the number and kinds of consumer goods they own, ranging from a television to a bicycle or car, and housing characteristics such as source of drinking water, toilet facilities, and flooring materials. These scores are derived using principal component analysis [36]. National wealth quintiles are compiled by assigning the household score to each usual (de jure) household member, ranking each person in the household population by their score, and then dividing the distribution into five equal categories.

Religion was recoded as 1 = Islam and 2 = others, residential status was recoded as 1 = urban and 2 = rural. Regions were provided in the survey as 1 = Barisal, 2 = Chittagong, 3 = Dhaka, 4 = Khulna, 5 = Rajshahi and 6 = Sylhet. To be noted, BDHS 2017–18 had eight regions namely Barisal, Chittagong, Dhaka, Khulna, Mymensingh Rajshahi, Rangpur and Sylhet. For analytical reasons Mymensingh was merged in Dhaka and Rangpur was merged in Rajshahi as these were divided at certain points after 2004.

### Statistical analysis

Descriptive analysis and bivariate analysis were conducted for a general characterization of the samples. Proportion test was used to reveal the significance level for the difference in the prevalence of stunting from 2004 to 2017–18. Logistic regression was used to find out the significant factors contributing to the prevalence of stunting among U5 children. Furthermore, fairlie decomposition technique was used to identify the crucial factors that contributed to the reduction of stunting. At first, the crude difference of stunting rates across the two samples were represented in the bivariate analysis; however to identify the difference and underlying causes of the decadal difference in the prevalence of stunting, the technique of decomposition was used identify and quantify inter-group differences. The original Blinder and Oxaca decomposition method is attributed to Blinder (1973) and Oaxaca (1973) who developed it to analyse continuous variables [37]. This technique, was extended and named “Fairlie decomposition” which is appropriate for binary models to decompose the decadal change in the prevalence stunting among children under age five years into contributions that can be attributed to different factors [33].

$$ {Y}^{t1}-{Y}^{t2}=\left[\sum \limits_{i=1}^{N^{t1}}\frac{F\left({X}_i^{t1}{\beta}^{t2}\right)}{N^{t1}}-\sum \limits_{i=1}^{N^{t2}}\frac{F\left({X}_i^{t2}{\beta}^{t2}\right)}{N^{t2}}\right]+\left[\sum \limits_{i=1}^{N^{t1}}\frac{F\left({X}_i^{t1}{\beta}^{t1}\right)}{N^{t1}}-\sum \limits_{i=1}^{N^{t1}}\frac{F\left({X}_i^{t1}{\beta}^{t2}\right)}{N^{t1}}\right] $$

Where Y is the dependent variable (stunting) at time t1 (2004) and t2 (2017–18), *N*^*J*^ is the sample size for time t, *X*^*J*^ is the row vector of average values of the independent variable and *β*^*J*^ is the vector of coefficient estimates for time t. This method of decomposition allows us to quantify the absolute contribution of factors explaining the decadal change (2004 to 2017–18) in the probability of U5 Bangladeshi children being stunted. Further, using mean variance inflation factor (VIF), we found no evidence of multicollinearity among the exaplantory variables. STATA 14 was used to carry out the analysis for the present study [38].

## Results

### Socio-economic and demographic profile of the mothers (Table 1)

The proportion of women who got married at age 18 or older increased from 39.8 to 56.8% between 2004 and 2017–18. There was a increase in the women’s educational status (30 percentage point) during these two survey periods. Similarly, the proportion of working women also increased from 17.6 to 42.4% between 2004 and 2017–18. There was a decline of low BMI women (from 38.4 to 15.3%). Conversely, the use of protected water declined slightly (from 96.7 to 85.4%) whereas the percentage of improved toilet facility increased from 56.7% to 57.8% between two survey periods.

**Table 1:** Socio-economic and demographic profile of study population in Bangladesh

Background characteristics	2004		2017–18	
	Sample	Percentage	Sample	Percentage
**Mother’s characteristics**				
**Age at first birth**				
Less than 18 years	3836	60.2	3589	43.2
18 years or more	2539	39.8	4723	56.8
**Educational status**				
Not educated	2396	37.6	594	7.2
Educated	3979	62.4	7718	92.8
**Working status**				
Not working	5256	82.5	4786	57.6
Working	1119	17.6	3526	42.4
**Low BMI**				
No	3930	61.7	7041	84.7
Yes	2445	38.4	1271	15.3
**Father’s characteristics**				
**Educational status**				
Not educated	2556	40.1	1216	14.6
Educated	3819	59.9	7096	85.4
**Working status**				
Not working	79	1.2	184	2.2
Working	6296	98.8	8128	97.8
**Child’s characteristics**				
**Children age (in months)**				
0–23	2503	39.3	3452	41.5
23–47	2605	40.9	3244	39.0
48–59	1267	19.9	1616	19.4
**Sex**				
Male	3237	50.8	4335	52.2
Female	3138	49.2	3977	47.8
**Birth order**				
1	1830	28.7	3185	38.3
2	1635	25.6	2683	32.3
3	1162	18.2	1408	16.9
4 and above	1748	27.4	1036	12.5
**Household characteristics**				
**Protected water source**				
No	212	3.3	1215	14.6
Yes	6163	96.7	7097	85.4
**Improved toilet facility**				
No	2759	43.3	3509	42.2
Yes	3616	56.7	4803	57.8
**Wealth Index**				
Poorest	1604	25.2	1782	21.4
Poorer	1323	20.8	1690	20.3
Middle	1244	19.5	1568	18.9
Richer	1150	18.0	1653	19.9
Richest	1053	16.5	1619	19.5
**Religion**				
Islam	5876	92.2	7644	92.0
Others	499	7.8	668	8.0
**Residential status**				
Urban	1253	19.7	2241	27.0
Rural	5122	80.4	6071	73.0
**Region**				
Barisal	379	5.9	461	5.6
Chittagong	1400	22.0	1744	21.0
Dhaka	1967	30.9	2814	33.9
Khulna	684	10.7	767	9.2
Rajshahi	1417	22.2	1848	22.2
Sylhet	528	8.3	678	8.2
Total	6375	100.0	8312	100.0

### Prevalence of stunting among children under five years (Table 2)

Results revealed that there was a significant decline (from 49.8% to 30.7%) in the prevalence of stunting among children age below 5 years in Bangladesh bewteen two survey periods (2004 and 2017–18). The decline in the prevalence of stunting was observed in all background characteristics (included mother, husband, child and household’s characteristics).

**Table 2:** Prevalence of stunting among children aged under five years by background characteristics in Bangladesh

Background characteristics	2004 (%)	2017–18 (%)	Difference	***p***-value
**Mother’s characteristics**				
**Age at first birth**				
Less than 18 years	51.7	33.5	−18.2	< 0.001
18 years or more	47.0	28.6	−18.4	< 0.001
**Educational status**				
Not educated	57.5	42.9	−14.6	< 0.001
Educated	45.2	29.8	−15.5	< 0.001
**Working status**				
Not working	49.5	28.3	−21.2	< 0.001
Working	51.5	34.0	−17.6	< 0.001
**Low BMI**				
No	45.6	29.0	−16.6	< 0.001
Yes	56.7	40.7	−16.0	< 0.001
**Father’s characteristics**				
**Educational status**				
Not educated	56.5	43.6	−12.9	< 0.001
Educated	45.4	28.5	−16.9	< 0.001
**Working status**				
Not working	44.0	34.0	−10.0	< 0.001
Working	49.9	30.6	−19.3	< 0.001
**Child’s characteristics**				
**Children age (in months)**				
0–23	39.0	26.9	−12.0	< 0.001
23–47	58.6	36.1	−22.5	< 0.001
48–59	53.6	28.4	−25.2	< 0.001
**Sex**				
Male	50.6	30.8	−19.8	< 0.001
Female	49.1	30.7	−18.4	< 0.001
**Birth order**				
1	47.9	28.7	−19.1	< 0.001
2	45.5	28.5	−17.1	< 0.001
3	48.2	32.0	−16.2	< 0.001
4 and above	57.0	41.0	−16.1	< 0.001
**Household characteristics**				
**Protected water source**				
No	55.0	28.7	−26.3	< 0.001
Yes	49.7	31.1	−18.6	< 0.001
**Improved toilet facility**				
No	56.9	34.3	−22.6	< 0.001
Yes	44.4	28.1	−16.3	< 0.001
**Wealth Index**				
Poorest	60.8	40.3	−20.5	< 0.001
Poorer	54.5	37.3	−17.2	< 0.001
Middle	49.5	30.1	−19.4	< 0.001
Richer	47.6	26.7	−21.0	< 0.001
Richest	30.2	17.2	−13.0	< 0.001
**Religion**				
Islam	50.4	30.9	−19.5	< 0.001
Others	43.5	28.8	−14.7	< 0.001
**Residential status**				
Urban	44.2	25.3	−18.8	< 0.001
Rural	51.2	32.7	−18.6	< 0.001
**Region**				
Barisal	55.3	32.8	−22.5	< 0.001
Chittagong	52.4	32.5	−19.9	< 0.001
Dhaka	51.3	27.9	−23.4	< 0.001
Khulna	40.6	25.6	−15.0	< 0.001
Rajshahi	47.2	30.6	−16.5	< 0.001
Sylhet	52.9	42.4	−10.5	< 0.001
Total	49.8	30.7	−19.1	< 0.001

*p-value based on proportion test*

**Fig. 1:**
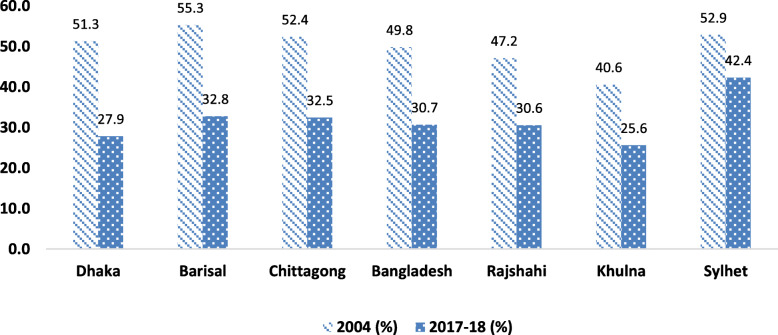
Change in the prevalence of stunting among children aged under five years by regions of Bangladesh

Figure 1 revealed that overall the prevalence of stunting among children had declined by 19 percentage point. Moreover, the highest decline was observed in Dhaka followed by Barisal and Chittagong.

### Results from logistic regression analysis for stunting among children below five years (Table 3) 2017–18

Estimates from logistic regression analysis showed that the likelihood of stunting among under-five children was 46% significantly less in 2017–18 than 2004 [AOR: 0.54; CI: 0.49–0.59]. Similarly, the odds of stunting were 29% significantly less among children whose mother did not have low BMI compared to their counterparts [AOR: 0.71; CI: 0.66–0.78]. The likelihood of stunting was 18% less likely to occur among children whose fathers were educated than those whose fathers did not receive any formal education [AOR: 0.82; CI: 0.75–0.90]. Moreover, children in the age range of 23–47 months [AOR: 1.85; CI: 1.71–2.01] and 48–59 months [AOR: 1.35; CI: 1.22–1.49] had 85% and 35% higher likelhood of being stunted with reference to children in the age of 0–23 months. Similarly, children’s from birth order of 4 or more were 24% significnalty more likely to be stunted in reference to children from birth order as one [AOR: 1.24; CI: 1.11–1.38]. The odds of stunting was lower among those households who had improved toilets than those who did not used [AOR: 0.92; CI: 0.84–1.06]. The odds of stunting among children decreased with the increase of wealth of the household. Moreover, the likelihood of stunting was 22% less among children who belonged to other religion group compared to children who belonged to Islam religion [AOR: 0.88; CI: 0.77–1.08]. Sylhet region had 30% higher likelihood of stunting among children with reference to Barisal region [AOR: 1.30; CI: 1.12–1.51].

**Table 3:** Logistic regression estimates for stunting aged under five by background characteristics in Bangladesh

Background characteristics	AOR(95% CI)
**Year**	
2004	Ref.
2017–18	0.54*(0.49,0.59)
**Mother’s characteristics**	
**Age at first birth**	
Less than 18 years	Ref.
18 years or more	0.95(0.88,1.02)
**Educational status**	
Not educated	Ref.
Educated	0.92(0.83,1.02)
**Working status**	
Not working	Ref.
Working	1.04(0.96,1.13)
**Low BMI**	
No	0.71*(0.66,0.78)
Yes	Ref.
**Father’s characteristics**	
**Educational status**	
Not educated	Ref.
Educated	0.82*(0.75,0.90)
**Working status**	
Not working	Ref.
Working	1.04(0.8,1.37)
**Child’s characteristics**	
**Children age (in months)**	
0–23	Ref.
23–47	1.85*(1.71,2.01)
48–59	1.35*(1.22,1.49)
**Sex**	
Male	Ref.
Female	1.00(0.93,1.08)
**Birth order**	
1	Ref.
2	0.96(0.87,1.05)
3	1.01(0.91,1.13)
4 and above	1.24*(1.11,1.38)
**Household characteristics**	
**Protected water source**	
No	Ref.
Yes	1.00(0.88,1.14)
**Improved toilet facility**	
No	Ref.
Yes	0.92*(0.84,1.06)
**Wealth Index**	
Poorest	Ref.
Poorer	0.93(0.83,1.04)
Middle	0.75*(0.67,0.85)
Richer	0.67*(0.59,0.75)
Richest	0.35*(0.3,0.4)
**Religion**	
Islam	Ref.
Others	0.88*(0.77,1.08)
**Residential status**	
Urban	Ref.
Rural	0.93(0.86,1.02)
**Region**	
Barisal	Ref.
Chittagong	1.09(0.95,1.25)
Dhaka	0.94(0.82,1.08)
Khulna	0.75*(0.64,0.87)
Rajshahi	0.77*(0.67,0.88)
Sylhet	1.30*(1.12,1.51)

*p < 0.05; *AOR* Adjusted Odds Ratio, *CI* Confidence Interval, *Ref* Reference category

### Estimates from decomposition analysis for stunting among children (Table 4)

Table 4 provides the estimates of decomposition analysis for the change in stunting among U5 children from 2004 to 2017–18 in Bangladesh. Fairlie decomposition analysis was used to find out the factors which contributed towards the increment or decrement of the prevalence of stunting between 2004 to 2017–18. Overall, selected variables could explain 50.6% of the decrease in the prevalence of stunting. A contribution with a positive sign shows that a particular factor has increased the prevalence of stunting between 2004 and 2017–18. However, if the contribution is negative, that means a decline in the prevalence of stunting was observed. Mother’s characteristics (29.4%) were the primary contributors explaning the change in the prevalence of stunting from 2004 to 2017–18 followed by father’s characteristics (9%). Moreover, within the mother’s characteristics, body mass index of mother (13.7%) followed by their educational status (10.5%) contributed most towards the change in the stunting from 2004 to 2017–18. Among father’s characteristics, educational status of father (9%) was the prime factor towards the change in stunting among children age below 5 year. Birth order was the major contributor (3.9%) towards the change in the prevalence of stunting among children.

**Table 4:** Decomposition estimates for stunting among children aged under five in Bangladesh, 2004 to 2017–18

Background characteristics	Coef.	Std. error	***p***-value	% Contribution
**Mother characteristics**				
**Age at first birth**				
Less than 18 years	Ref.			
18 years or more	0.003	0.002	0.063	1.6
**Educational status**				
Not educated	Ref.			
Educated	0.020	0.004	0.000	10.5
**Working status**				
Not working	Ref.			
Working	0.007	0.002	0.006	3.6
**Low BMI**				
No	Ref.			
Yes	0.026	0.003	0.000	13.7
**Father characteristics**				
**Educational status**				
Not educated	Ref.			
Educated	0.017	0.003	0.000	9.0
**Working status**				
Not working	Ref.			
Working	0.000	0.000	0.969	0.0
**Child characteristics**				
**Children age (in months)**				
0–23	Ref.			
23–47	0.000	0.000	0.000	0.2
48–59	0.001	0.000	0.000	0.6
**Sex**				
Male	Ref.			
Female	0.000	0.000	0.971	0.0
**Birth order**				
1	Ref.			
2	0.001	0.001	0.117	0.6
3	0.000	0.000	0.721	0.0
4 and above	0.007	0.002	0.000	3.9
**Household characteristics**				
**Protected water source**				
No	Ref.			
Yes	0.004	0.002	0.003	2.3
**Improved toilet facility**				
No	Ref.			
Yes	0.001	0.000	0.155	0.3
**Wealth Index**				
Poorest	Ref.			
Poorer	0.000	0.000	0.694	−0.1
Middle	−0.001	0.000	0.405	−0.5
Richer	0.001	0.000	0.000	0.6
Richest	0.008	0.001	0.000	4.0
**Religion**				
Islam	Ref.			
Others	0.000	0.000	0.201	0.1
**Residential status**				
Urban	Ref.			
Rural	0.000	0.001	0.952	0.0
**Region**				
Barisal	Ref.			
Chittagong	0.000	0.000	0.740	0.0
Dhaka	0.001	0.000	0.045	0.4
Khulna	0.001	0.000	0.005	−0.4
Rajshahi	0.000	0.000	0.143	0.2
Sylhet	0.000	0.000	0.563	0.0
				50.6
N (2004)	6003			
N (2018)	7849			
Predictive probability of being stunted in 2004 (p1)	0.498			
Predictive probability of being stunted in 2017–18 (p2)	0.307			
Difference (p1-p2)	0.191			
Total explained	0.097			

## Discussion

Since stunting replicates the accumulative outcome of socio-economic, health and nutritional drawbacks that vary over time [39, 40], the continuous evaluation of underlying factors may help boost the pace of its reduction. In spite of substantial achievements in reducing the prevalence rates of stunting among under-five children in Bangladesh through various intervention programs, the findings of this study with 30.7% children still being stunted with marked regional variations underscore the need for further improvement in the children’s nutritional status in the country.

In the present study, we found that children of age group 23–47 months, with higher birth order, born to mothers with low BMI, to mothers and fathers with no formal education and belonging to households with poor sanitation and poorest wealth index were significantly more likely to be stunted. Results from binary logistic regression show that as children grow up they become more likely to be stunted. Similarly, under five children who were most likely to be stunted belonged to the age of 23–47 months. And the result is consistent with recent studies in Bangladesh [16, 41, 42]**.** Higher prevalence of stunting among children who aged 2 year and older can be explained by inapropriated or untimely initiation of complementary feeding and lack of dietary diversity and nutritional knowledge that are found to be major risk factors of stunting [43, 44]. Furthermore, the finding that fifth or higher order birth is related to a greater prevalence of stunting is similar to another study in Bengladesh [45], and is so probably due to lesser time to extend care equally to each of the children. It is also noted that having a higher number of siblings in a household may cause growth retardation in children due to competition for limited available nutritional resources [46]. However, the decadel decline in children with higher birth order possibly due to declining fertility and growing number of small-sized families has significantly contributed to the observed decrease in childhood stunting in the present study.

Studies in low-and-middle income countries reveal that the child is inherited with how he/she was cared both in the womb and during childhood and there exists an intergenerational cycle of health and nutrition [47–49]. The study result suggests that healthier mothers have lower risk of having stunted children. The finding that the maternal higher BMI as a protective predictor for childhood stunting is consistent with previous studies [50–52]. It is argued that with good nutritional status, mothers can confirm better breastfeeding and react to pregnancy-related stressors effectively [42, 53]. In addition, current study found that mother’s low BMI had a significant and largest contribution to decadel decrease in childhood stunting compared to all other factors. It reflects the findings of recent studies in Bengladesh on the success of delivering multiple interventions for maternal nutrition and health [54–56].

Education of parents is found to be another significant contributer to the decrease in the prevalence of stunting among the children below 5 years, indicating the improvements in overall socioeconomic indicators of the general population in the country. Again, education being a significant predictor shows that higher levels of education can act as a preventive factor against child stunting. Thus, children may become stunted due to their mothers’ or fathers’ lower educational status and lack of sources of knowledge on child nutrition. Evidence suggests that education always plays a positive role in health and diseases [57, 58]. Studies conducted in low and middle income countries also reveal that educated mothers tend to be older at their first birth and are more knowledgeable about health, nutrition, hygiene and care practices for children [59–61]. In addition, a recent study shows that maternal awareness on childfeeding practices also led to improved nutritional status of young children [62]. Educated parents can further contribute to a better household economic status that is supportive of providing sufficient nutritional diet to their children. Additionally, they tend to have have greater access to media that make them better able to use available health-care facilities and more adept at keeping their environment clean [52, 63].

Furthermore, nutritional status of children is considered as an indicator that reflects the household economic condition. This study also shows that children from households with poorest wealth index are most likely to be stunted in consistence with previous studies conducted in other countries [42, 61, 64, 65]. This can be attributed to the fact that households with higher socioeconomic status may have more ability to allocate necessary resources regarding nutrition for their children. Also, reasonable allocation of resources may improve their children’s health conditions by minimizing multiple health risks. Consistent with past studies [15, 60, 66], access to improved toilet facility in the households was found as a significant determinant of stunting among under-five children. Poor sanitation facilities have negative impact on child’s nutritional status through pathways such as loss of appetite, loss of host tissues and maldigestion, resulting in a vicious cycle of growth faltering in childhood [67]. It is also documented that an adverse environment may cause differences in the metabolism of micronutrients in children and potentially limit their ability to develop into healthy adults [30, 68].

Finally, we observed large regional variations in the prevalence of stunting among under-five children, where highest prevalence was found in the Sylhet administrative division. Although the Sylhet division is established in previous studies as poorest performing region in most of the health-related indicators including child stunting [52, 69], the situation still remains the same. Thus, region specific studies are warranted for seeking explanations of why the stunting of Bengladeshi children varies across different divisions.

This study has certain limitations. Firstly, the study was based on cross-sectional data, which failed to establish a casual relationship. Importantly, the study assumes that stunting is due primarily to nutrition and any of the nutritional variables were not included in the analysis. Another major limitation is that this study has particularly focused only some selected factors. The factors, such as duration of breast feeding, household size, use of iodized salt and dietary diversity, among others that may also affect stunting among children were not analysed.

The strenths of this study the results identified the most vital risk factors of stunting, which will add to the available literature on the association of socioeconomic and demographic variables and parents’ characteristics with stunting of under five children in Bengladesh. Besides, the survey data used in the present analysis was based on a two-stage stratified sampling of households and was designed to generate representative results for the country as a whole and for each of the seven administrative divisions.

## Conclusion

The results of the study strongly highlight the importance of taking steps to make people educated and to reduce household wealth inequality to improve nutritional status of children. In order to achieve further reduction in stunting among under-five children in Bangladesh, target interventions that would benefit the socioeconomically backward population are required. Results also suggest that the policymakers should develop an effective program to improve the maternal education and health and increase awareness of parents about the standard children’s height and weight according to their age and gender. Despite making gains in macro level health and development indicators, there is a need to address the underlying issues of child malnutrition in Bangladesh. Therefore, policymakers need to revisit and expand current mother-child specific programs and interventions targeting vulnerable regions and households. Also, government may take necessary steps to make women of reproductive age aware about adverse effects of higher birth order in their later ages and its adverse effects on child health.

## Data Availability

The study utilizes a secondary source of data that is freely available in the public domain through, https://dhsprogram.com/data/dataset/Bangladesh_Standard-DHS_2017.cfm?flag=1

## Abbrevations

BDHS: Bangladesh Demographic and Health Survey
NIPORT: National Institute for Population Research and Training
EA: Enumeration Area
PSU: Primary Sampling Unit
BBS: Bangladesh Bureau of Statistics
SD: Standard Deviation
WHO: World Health Organization
AOR: Adjusted Odds Ratio
CI: Confidence Interval
